# Disseminated tuberculosis complicating Bacillus Calmette–Guérin (BCG) vaccine as only presentation of severe combined immunodeficiency (SCID): A report of 3 cases

**DOI:** 10.5339/qmj.2023.sqac.9

**Published:** 2023-11-19

**Authors:** Karmel Omer, Gufran Algaly, Omaima Abdelmajeed Mohamed Salih

**Affiliations:** ^1^Medical officer, Tropical Disease hospital; ^2^Emergency Medicine Resident, Hamad Medical Corporation Email: GALGALY@hamad.qa; ^3^Departments of Pediatrics, Faculty of Medicine, Omdurman Islamic University, Pediatric Tropical consultant, clinical immunologist, Tropical diseases pital

**Keywords:** SCID, Primary immunodeficiency, Disseminated Tuberculosis

## Abstract

Introduction: Severe combined immunodeficiency disease (SCID) is a rare primary immunodeficiency disease, usually manifest in the first six months of life with failure to thrive, oral thrush, recurrent respiratory infection, and chronic diarrhea.

Case presentation: In three male patients, we describe an unusual presentation of SCID. They are an outcome of consanguineous marriage; all received the BCG vaccine at birth. All three cases presented with regional lymphadenopathy at three months, progressing to generalized lymphadenopathy treated with anti-tuberculous. The first and second cases were twins. The first had an uneventful history until 33 months when he developed multiple Suppurative Tuberculous lymphadenitis confirmed by biopsy. The second and the third cases were diagnosed with Disseminated Tuberculosis at 24 months as they developed fever, anemia, weight loss, tuberculous peritonitis, and lymphadenopathy confirmed by biopsy. After investigations, the first case was diagnosed as CD4, CD16 lymphopenic SCID, the second one as CD4, CD8, CD19, CD16 lymphopenic SCID with hypogammaglobulinemia and the third case as CD3, CD4, CD8 lymphopenic SCID with hypogammaglobulinemia. They received anti-Tuberculous medications, prophylactic Trimethoprim/Sulfamethoxazole, and Immunoglobulin infusion. When writing this abstract, the patients were alive and had no other bacterial, viral, or fungal infections. The twins are three years old, and the third case is 30 months old.

Conclusion: SCID may not exhibit the classical manifestation of recurrent infections. It may present only as a complication of the BCG vaccine, alarming to maintain high susceptibility in such patients, especially in a developing country, specifically in Sudan, where the BCG vaccine is usually given at birth.

